# Inflammatory Biomarkers in Heart Failure: Clinical Perspectives on hsCRP, IL-6 and Emerging Candidates

**DOI:** 10.1007/s11897-025-00710-3

**Published:** 2025-11-06

**Authors:** Berkan Kurt, Konstantin Rex, Martin Reugels, Christopher B. Fordyce, Marat Fudim, Abhinav Sharma, Martin Berger, Nikolaus Marx, Katharina Marx-Schütt, Florian Kahles

**Affiliations:** 1 Department of Internal Medicine I - Cardiology, University Hospital Aachen, Aachen, Germany; 2 Centre for Cardiovascular Innovation, Division of Cardiology, Vancouver General Hospital, University of British Columbia, Vancouver, BC Canada; 3 Duke Clinical Research Institute, Division of Cardiology, Department of Medicine, Duke University School of Medicine, Durham, NC USA; 4 Division of Cardiology, Research Institute of the McGill University Health Centre, McGill University, Montreal, QC Canada

**Keywords:** Biomarker, Inflammation, HsCRP, IL-6, Prognosis, Risk stratification, Prevention

## Abstract

**Purpose of Review:**

Heart failure (HF) remains a leading cause of morbidity and mortality worldwide. Increasing evidence highlights that systemic low-grade inflammation is a key pathophysiological driver of HF. This review seeks to examine the diagnostic and therapeutic relevance of inflammatory biomarkers – specifically interleukin-6 (IL-6) and high-sensitivity C-reactive protein (hsCRP) – and evaluate their potential for improving risk stratification and enabling personalized treatment approaches in HF.

**Recent Findings:**

IL-6 and hsCRP have emerged as important markers of residual inflammatory risk in HF. Elevated levels of these biomarkers are associated with increased risk of incident HF and adverse outcomes in established disease. While hsCRP is as a downstream marker of inflammation with no causal involvement, Mendelian randomization studies support a causal role of IL-6 signaling in the development of HF and coronary artery disease. Recent and ongoing clinical trials support the concept of targeting inflammatory pathways as a therapeutic strategy in selected HF populations.

**Summary:**

Inflammatory biomarkers, particularly IL-6 and hsCRP, are promising tools for advancing precision medicine in HF by improving individual risk assessment and guiding anti-inflammatory interventions. Further large-scale studies are needed to validate the integration of inflammatory biomarkers into clinical algorithms for HF and explore their potential role in future guideline recommendations and personalized prevention strategies.

## Introduction

Heart failure (HF) affects approximately 64 million individuals worldwide and is a clinical syndrome characterized by symptoms and signs caused by underlying functional or structural myocardial impairment limiting the heart’s ability to maintain adequate cardiac output under physiological demands [1–3]. Among patients aged 65 and older it remains the leading cause of hospitalization and is a substantial burden to economic systems, driven by acute decompensation of pre-existing HF [2]. Despite best medical efforts, including guideline-recommended medical therapy, morbidity and mortality in HF patients remain high [2]. Current therapeutic approaches, including Renin-Angiotensin-Aldosterone System inhibitors (RAAS inhibitors), beta-adrenergic blockers, Sodium-Glucose Co-Transporter-2 inhibitors (SGLT-2i) and Mineralocorticoid Receptor Antagonists (MRAs) aim to modulate the neurohumoral axis, attenuate myocardial oxygen demand, decrease adverse cardiac remodeling and enhance myocardial metabolic efficiency [3, 4]. However, addressing residual risk attributed to chronic low-grade inflammation even after optimal treatment has not been integrated in guideline-directed management of HF yet. Identifying patients with residual inflammatory risk observed in patients with HF with assessment of inflammatory biomarkers is crucial to enhance risk stratification, enable targeted interventions and guide therapeutic decision-making [5]. This review discusses current evidence and emerging perspectives on the use of inflammatory biomarkers in patients with HF.

### Inflammation in Cardiovascular Disease

Systemic low-grade inflammation plays a critical role in the pathophysiology of cardiovascular disease (CVD), including HF and atherosclerotic cardiovascular disease (ASCVD) [6]. Multiple triggers enhance systemic low-grade inflammation, including established cardiovascular (CV) risk factors and comorbidities such as obesity, dyslipidemia, smoking, type 2 diabetes mellitus (T2DM), hypertension, chronic kidney disease (CKD) and chronic inflammatory or autoimmune disorders [7–11]. In the past two decades, there have been various trials investigating the inflammatory hypothesis for development of CVD and anti-inflammatory therapeutic strategies have gained significant attention in this context. One of the landmark studies has been the “Justification for the Use of Statins in Prevention: an Intervention Trial Evaluating Rosuvastatin” (JUPITER) trial, showing that rosuvastatin led to reduction of major adverse cardiovascular events (MACE) in apparently healthy individuals with low-density lipoprotein cholesterol (LDL-C) levels < 130 mg/dL and concomitant residual inflammatory risk defined by high-sensitivity C-reactive protein (hsCRP) levels ≥ 2 mg/L [12]. The “Canakinumab Anti-inflammatory Thrombosis Outcome Study” (CANTOS) published in 2017 further validated the inflammatory hypothesis by demonstrating that canakinumab, a therapeutic monoclonal antibody targeting interleukin-1β, reduced CV events in patients with previous myocardial infarction (MI) [13]. In addition, the “Low-Dose Colchicine 2” (LoDoCo2) trial, highlighted the risk reduction for cardiovascular events achieved by targeting the nucleotide-binding domain-like receptor protein 3 (NLRP3) inflammasome in ASCVD, emphasizing the therapeutic potential of anti-inflammatory strategies in CVD [14]. Recent evidence however has challenged our current understanding of the inflammatory hypothesis, particularly in acute coronary syndrome. The “CoLchicine and spironolactonE in patients with ST elevation myocARdial infarction/SYNERGY Stent Registry – Organization to Assess Strategies for Ischemic Syndromes 9” (CLEAR-SYNERGY (OASIS 9)) study has demonstrated that in patients, treatment with colchicine, when started soon after MI and continued for a median of 3 years, did not reduce the incidence of the composite primary outcome (death from cardiovascular causes, recurrent MI, stroke, or unplanned ischemia-driven coronary revascularization). These results highlight the importance of careful patient selection for future therapeutic anti-inflammatory strategies [15].

### Heart Failure Subtypes and Pathophysiology

The European Society of Cardiology (ESC) classifies HF into three categories based on left ventricular ejection fraction (LVEF): HF with reduced ejection fraction (HFrEF, LVEF ≤ 40%), mildly reduced ejection fraction (HFmrEF, LVEF 41–49%), and preserved ejection fraction (HFpEF, LVEF ≥ 50%) [3, 4].

Ischemic heart disease (IHD) represents the most common cause of HF followed by hypertensive heart disease and further etiologies, such as valvular heart disease or tachycardia-induced cardiomyopathy [1]. Despite differences in pathophysiology, all subtypes share common clinical features and symptoms like dyspnea, fatigue or congestion, typically accompanied by elevated natriuretic peptides such as N-terminal pro-B-type natriuretic peptide (NT-proBNP). The diagnosis of HFpEF further requires evidence of cardiac structural and/or functional abnormalities, such as presence of LV diastolic dysfunction or signs of elevated LV filling pressures [4]. With improvements in medical therapy for IHD and an aging population, the prevalence of HFpEF is rising and expected to become the most common subtype of HF in the near future [2, 16].

The pathophysiological mechanisms of HF subtypes vary according to underlying etiologies and associated risk factors. HFrEF is predominantly associated with IHD, in which myocardial injury is primarily caused by atherothrombosis and followed by reduced coronary blood-flow. This results in cardiomyocyte necrosis leading to scar formation, impaired contractility, increased intracardial filling pressures and systemic congestion which are ultimately followed by decreased cardiac output and inadequate systemic oxygen delivery [1, 17]. HFrEF is typically accompanied by classic cardiovascular risk factors such as smoking, dyslipidemia, T2DM, and hypertension [1, 18–21]. In contrast, risk factors for HFpEF typically differ in emphasis and are characterized by different pathophysiological mechanisms, a diverse range of clinical phenotypes and associations with female sex and advanced age. HFpEF is frequently observed in patients with comorbidities such as obesity, T2DM, CKD, atrial fibrillation and chronic obstructive pulmonary disease (COPD) [3, 22]. Especially development of HFpEF and progression is closely linked to systemic inflammation [5, 23–25], which will be discussed in the following sections.

### The Role of Inflammation in Heart Failure Pathogenesis

**Fig. 1:**
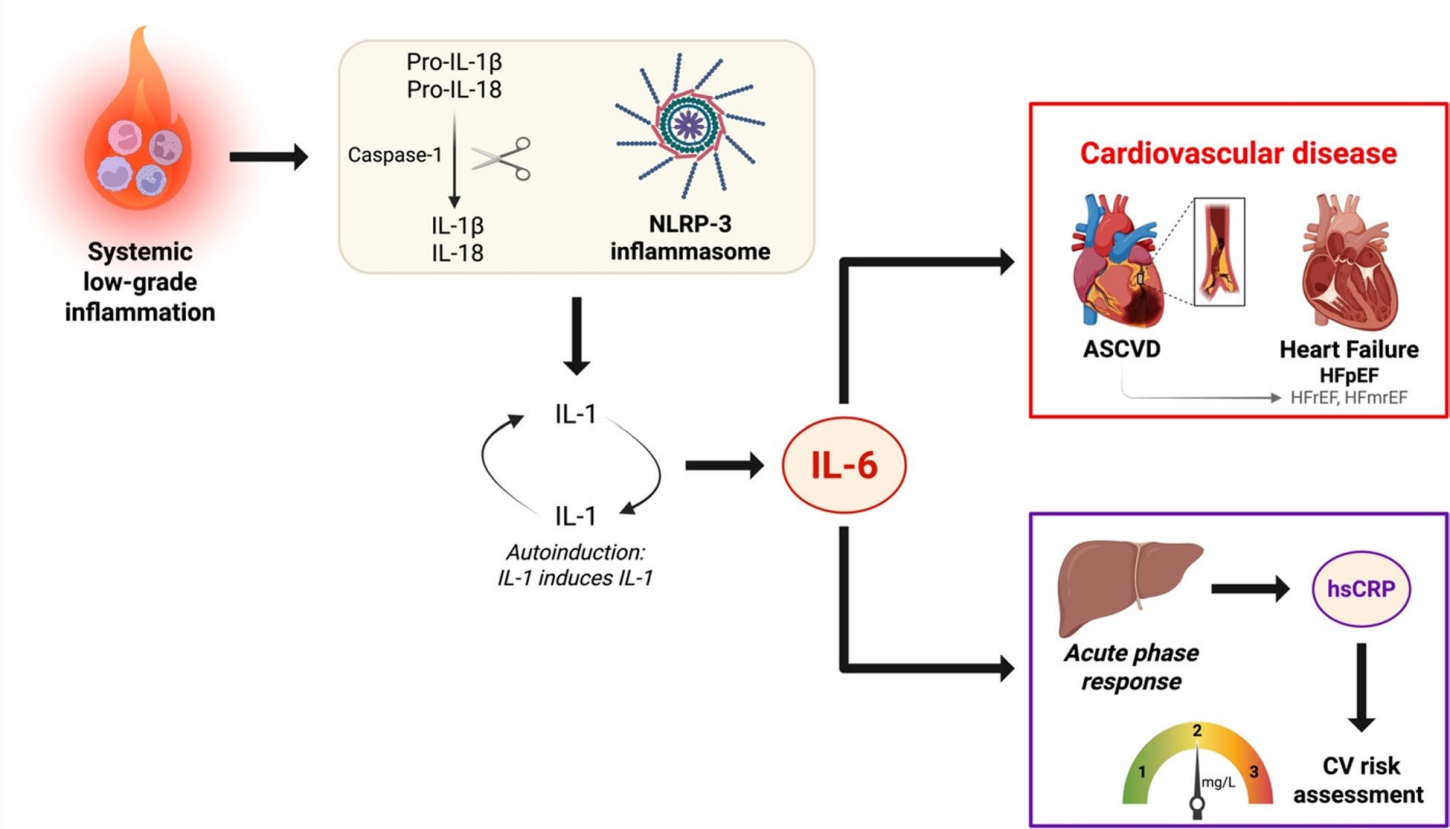
**The pathophysiology of systemic low-grade inflammation and CVD**. Schematic overview of the inflammatory cascade linking systemic low-grade inflammation to IL-6–driven cardiovascular disease and hsCRP-based risk assessment. Abbreviations: ASCVD = Atherosclerotic cardiovascular disease; CV: cardiovascular; Gal-3 = Galectin-3; Gal-9 = Galectin-9; GDF-15 = Growth differentiation factor 15; HFmrEF = Heart failure with mildly reduced ejection fraction; HFpEF = Heart failure with preserved ejection fraction; HFrEF = Heart failure with reduced ejection fraction; hsCRP = High-sensitivity C-reactive protein; IL-1 = Interleukin-1; IL-1β = Interleukin-1 beta; IL-6 = Interleukin-6; IL-18 = Interleukin-18; NLRP3 = Nucleotide-binding domain-like receptor protein 3; sST2 = Soluble suppression of tumorigenicity-2; TNF-α = Tumor necrosis factor alpha (Figure created with BioRender.com; adapted from Ridker [39] and Libby [29])

The cardiometabolic comorbidities of HFrEF and HFpEF are associated with systemic inflammation, which contributes to the development and progression of HF but is also linked to higher disease burden in patients with advanced HF, suggesting a vicious circle in which inflammation initiates and sustains cardiac dysfunction and adverse remodeling [26–28]. Central to the inflammatory cascades that drive HF-development are cytokines including Tumor Necrosis Factor-α (TNF-α), interleukin-6 (IL-6) and acute-phase proteins (APP) (Fig. 1). Following NLRP3 inflammasome- and subsequent IL-1β activation, IL-6 is synthesized during the early stages of inflammation and orchestrates further pro-inflammatory responses [29]. These cytokines are secreted by various cell types (e.g. leucocytes, endothelial cells, cardiomyocytes) and lead to stimulation of hepatocytes to produce APPs such as C-reactive protein (CRP) [30, 31].

Among pro-inflammatory cytokines and APPs studied for causal relationships in CVD, IL-6 is supported by the most consistent genetic evidence. Mendelian randomization studies have suggested a driving role for IL-6 signaling in the development of coronary artery disease, a common underlying etiology of HF [1, 32, 33]. More recent data have extended this association to HF itself, showing that genetic variants in IL-6 associated with reduced inflammatory signaling are linked to a lower risk of incident HF [34]. In contrast, despite its widespread use as a marker of residual inflammatory risk, no evidence currently suggests that CRP is causally involved in the pathogenesis or progression of CVD [5, 35–38]. These findings support a central role of IL-6 in the pathogenesis of atherosclerotic and myocardial disease.

**Fig. 2:**
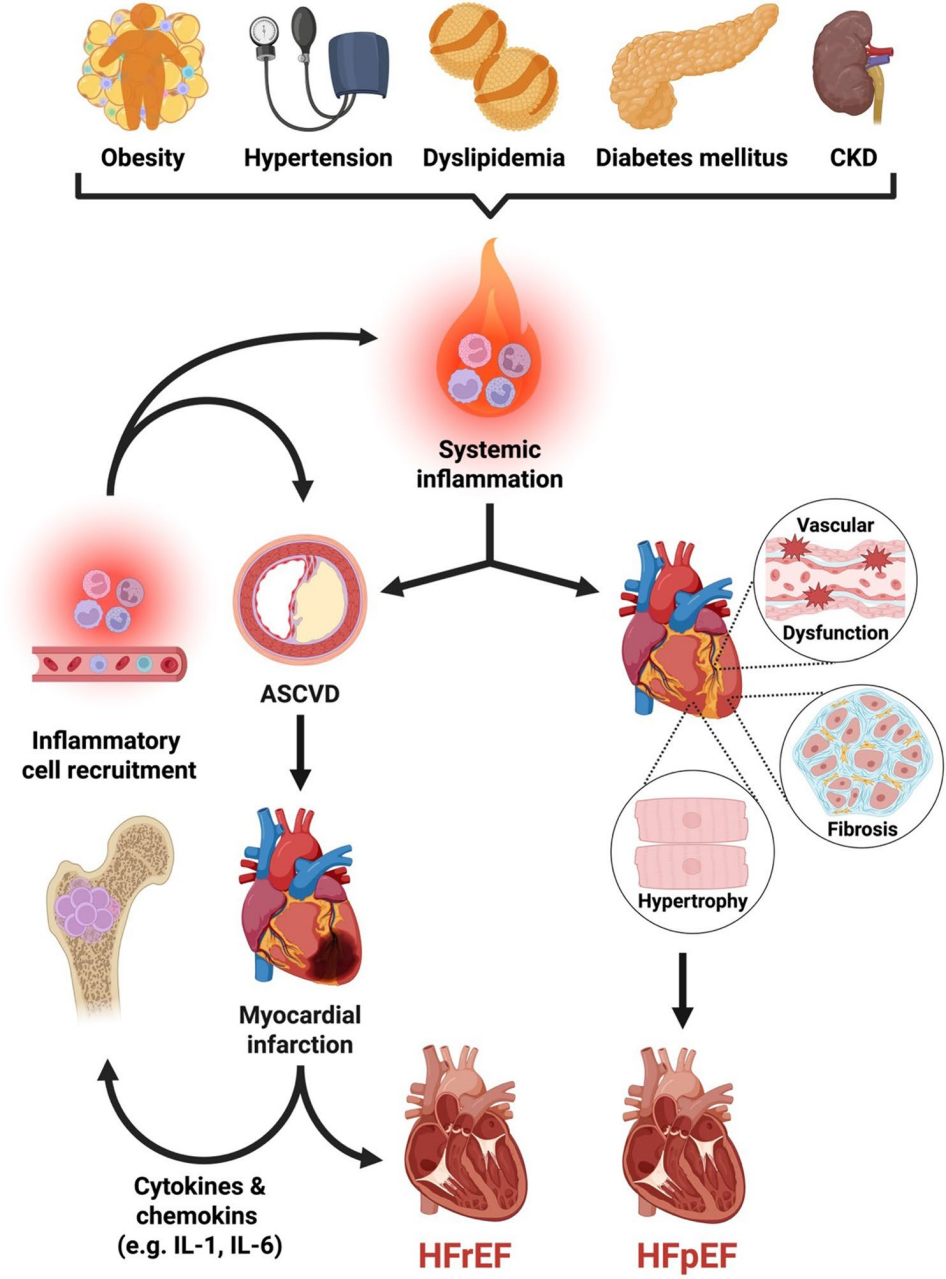
**Inflammation as a common link between cardiometabolic risk factors and heart failure subtypes**. Cardiometabolic comorbidities such as obesity, hypertension, dyslipidemia, diabetes, and CKD promote systemic low-grade inflammation, which contributes to ASCVD and HF. Inflammation drives pathophysiological mechanisms ultimately leading to HFpEF or HFrEF. Abbreviations: ASCVD = Atherosclerotic cardiovascular disease; CKD = Chronic kidney disease; HFpEF = Heart failure with preserved ejection fraction; HFrEF = Heart failure with reduced ejection fraction; IL-1 = Interleukin-1; IL-6 = Interleukin-6 (Figure created with BioRender.com; adapted from Pugliese et al. [53] and Libby et al. [54])

Inflammation contributes differently across HF subtypes (Fig. 2). Proinflammatory cascades play a central role in the development of HFpEF, often triggered by comorbidities such as obesity, diabetes, and chronic kidney disease, leading to endothelial dysfunction and myocardial fibrosis. Whereas in HFrEF, systemic low-grade inflammation not only contributes to the development of atherosclerosis and IHD but also emerges secondary following myocardial injury or ischemia, driving adverse remodeling through innate and adaptive immune activation [28]. Initial ischemic triggers of inflammation can be of chronic but also acute nature, in both of which cardiac macrophages and cardiomyocytes release pro-inflammatory cytokines and recruit neutrophils and monocytes from the bone marrow [31]. Following MI, immune responses with cytokine release aggravate myocardial injury and drive adverse cardiac remodeling with scar formation [31, 40–42]. Especially when dysbalanced and pro-inflammatory responses outweigh repair mechanisms in the setting of ischemia, myocardial healing is disturbed, ultimately leading to impairment of myocardial contractility and HFrEF [42].

For HFpEF, the interaction between inflammation and HF development has been extensively studied in experimental studies. Pro-inflammatory responses, mediated by IL-6 signaling, promote increased immune cell influx and oxidative stress, resulting in impaired nitric oxide (NO) bioavailability between coronary endothelial cells and cardiomyocytes [43, 44]. This contributes to the development of myocardial fibrosis and diastolic dysfunction, as seen in HFpEF [45–49]. In both, HFpEF patients and rat models, the reduction of NO-dependent signaling is associated with myocardial stiffness and hypertrophy [50]. Furthermore, studies in rats where IL-6 infusions have been performed showed reinforced development of concentric left ventricular hypertrophy and ventricular stiffness [51]. Adding to these findings, studies in IL-6 knockout mice demonstrated reduced left ventricular hypertrophy in response to pressure overload induced by transverse aortic constriction compared to wild-type mice [52]. These results strengthen the role of pro-inflammatory processes mediated by IL-6 in the development of diastolic dysfunction and HFpEF.

### Emerging Biomarkers in Heart Failure

Beyond their mechanistic role in the development and progression of HF, mediators of inflammation have intensively been studied as biomarkers for identifying individuals at risk for HF and for prognostic evaluation in established disease. Currently, routine measurement of inflammatory biomarkers is not recommended in HF guidelines. While the ESC endorses the assessment of hsCRP in the early diagnostic evaluation of presumed coronary artery disease in the 2024 Guidelines for the Management of Chronic Coronary Syndromes, there are no such recommendations in current ESC HF guidelines [3, 4, 55].

To be formally recognized, biomarkers are evaluated with specific criteria outlined by the Food and Drug Administration (FDA). Here, biomarkers are categorized into susceptibility/risk, diagnostic, monitoring, prognostic, predictive, pharmacodynamic and safety categories (“BEST categories”) based on their applicability in clinical practice and scientific evidence [56, 57]. Furthermore, three statistical criteria are used to assess a biomarkers utility: discrimination (a model’s ability to differentiate between individuals who will and will not experience events), calibration (the agreement between predicted and observed risks), and reclassification (the re-assignment of individuals into clinically more accurate risk categories). Such analyses are essential to determine whether biomarkers can optimize clinical decision-making and personalized prevention strategies [58].

Given their early described association with HF, inflammatory biomarkers may help address residual inflammatory risk and improve patient outcomes in HF by enhancing risk stratification and guiding anti-inflammatory therapies.

In 1990 Levine et al. reported elevated TNF-α levels in patients with chronic HF [59]. Dunlay et al. have demonstrated that increased TNF-α levels were associated with a 32% increase of all-cause mortality in 486 HF patients, irrespective of baseline LVEF [60]. Since then, numerous biomarkers have been identified to be associated with HF. Inflammation-related biomarkers such as growth differentiation factor-15 (GDF-15), soluble suppression of tumorigenicity-2 (sST2), Galectin-3 (Gal-3) and Galectin-9 (Gal-9) as well as hsCRP and IL-6, have been shown to predict new-onset HF and adverse CV events in HF patients [61–68] (Table 1). Their biological and pathophysiological background as well as their role in prediction of outcomes in human studies will be explored in the following.

**Table 1:** Inflammatory biomarkers in chronic heart failure

Biomarker	Mechanism/pathway	Predictive for HF incidence	Prognostic in HF	Stability	Guideline supported?	Example references
**IL-6**	Drives ASCVD, fibrosis, remodeling, endothelial dysfunction (**upstream cytokine)**	Yes	Yes	Moderate	No	[89–91, 95–102]
**hsCRP**	Reflects systemic inflammation; likely not causally related (**downstream APP)**	Yes	Yes	High	Risk-enhancing factor (ACC/AHA) [103], Risk modifier (CCS) [104], included in the SMART risk score for secondary prevention of ASCVD [105], Class IIa recommendation in the initial diagnostic workup in suspected CCS (ESC) [55]	[86, 88, 92–94, 97–99]
**GDF-15**	Promotes fibrosis, hypertrophy, inflammation (TGF-β cytokine)	Yes	Yes	Moderate	No	[73, 74, 78]
**sST2**	Promotes fibrosis; blocks IL-33/ST2L signaling (IL-1 family decoy receptor)	Yes	Yes	High	Previously (2017) recommended (class IIb) biomarker for prognosis or added risk stratification (ACC/AHA/HFSA) [106]	[76–78]
**Galectin-3**	Induces fibrosis and remodeling via macrophage activation	Yes	Yes	High	Previously (2017) recommended (class IIb) biomarker for prognosis or added risk stratification (ACC/AHA/HFSA) [106]	[81–83]

Overview of key inflammatory biomarkers in chronic HF, including mechanistic pathways, predictive and prognostic value, biomarker stability, and support by current clinical guidelines.

Abbreviations; *ACC* American College of Cardiology, *AHA* American Heart Association, *APP* Acute phase protein, *ASCVD* Atherosclerotic cardiovascular disease, *CCS* Chronic coronary syndrome, *Gal-3* Galectin-3, *GDF-5* Growth differentiation factor-5, *HFSA* Heart Failure Society of America; *hsCRP* High-sensitivity C-reactive protein, *IL-1* Interleukin-1, *IL-33* Interleukin-33, *sST2* Soluble suppression of tumorigenicity-2, *ST2L* Suppression of tumorigenicity-2 receptor – long isoform, *TGF-β* Transforming growth factor beta

GDF-15 shows diverse effects in CV-disease, influencing cardiomyocyte and endothelial function, hypertrophy, fibrosis and inflammation [61]. It regulates cardiomyocyte survival and proliferation through multiple signaling pathways, showing pro-apoptotic and pro-proliferative effects. In endothelial cells, GDF-15 modulates vascular function and contributes to angiogenesis and endothelial dysfunction by modification of NO release [69, 70]. GDF-15 is also linked to cardiac hypertrophy and shows pro- and anti-hypertrophic effects [71, 72]. While it is associated with cardiac fibrosis, the underlying mechanisms remain incompletely understood. As a member of the Transforming growth factor-β (TGF- β) family, GDF-15 is an inflammatory cytokine linked to chronic low-grade inflammation in HF [61]. A study assessing GDF-15 levels in humans showed an independent link to new-onset HF in 10,570 individuals from the Atherosclerosis Risk in Communities (ARIC) study. Moreover, the inclusion of GDF-15 in a multivariable risk prediction model provided incremental prognostic value for predicting new-onset HF [73]. Furthermore, in patients with HFrEF, trajectories of GDF-15 over 30 days were independently associated with an increased risk of death or hospitalization for HF (HHF) and declining exercise capacity [74].

As a member of the IL-1-receptor family, sST2 acts as a receptor that inhibits the cardioprotective IL-33/suppression of tumorigenicity-2 receptor – long isoform (ST2L) signaling pathway, promoting myocardial fibrosis and contributing to HF progression [65, 75]. In 3915 individuals without prior HF, elevated sST2 levels were linked to incident HF, CV mortality and improved risk discrimination as well as reclassification [76]. Moreover, in a study of 4268 HF patients, sST2 showed independent prognostic value, as increased sST2 levels were associated with an increase in all-cause mortality, CV-mortality and HHF [77]. Furthermore, in an analysis of 3428 individuals from the Framingham heart study sST2, GDF-15 and high-sensitivity troponin were independently associated with death, HF and cardiovascular events. A multimarker score including these three biomarkers along with B-type natriuretic peptide (BNP) and hsCRP significantly improved predictive capacity beyond established clinical risk markers [78].

Gal-3 is a beta-galactoside-binding lectin and involved in inflammation and development of fibrosis by accelerating migration and proliferation of macrophages as well as fibroblasts. It is increasingly recognized as a relevant biomarker for myocardial remodeling in HF [79]. Experimental studies have shown that addition of exogenous Gal-3 induced HF and promoted macrophage migration and fibroblast proliferation, resulting in excessive collagen deposition [80]. In human studies, Gal-3 levels were associated with an increased risk for incident HF, as well as all-cause mortality in 3353 participants [81]. These associations were supported by a meta-analysis published in 2023 demonstrating that elevated Gal-3 levels are linked to an increased risk of incident HF [82]. Furthermore, the predictive value of Gal-3 has been investigated in a cohort of 592 HF patients, where Gal-3 levels were independently associated with a 38% increased risk of all-cause mortality and HHF [83].

Gal-9 is an immune-checkpoint ligand that upregulates ß2-integrin expression and promotes neutrophil adhesion to endothelial cells and leukocyte recruitment. Elevated Gal-9 levels are associated with an increased risk of HHF and all-cause mortality in individuals with and without HF [68, 84].

Beyond the evaluation of individual biomarkers, recent research increasingly focusses on large-scale utilization of novel -omics approaches to improve risk stratification in HF. A recent study demonstrated that proteomics-based biomarker panels, in addition to clinical risk factors and NT-proBNP, improved prediction of HF incidence [85]. However, the complexity of these approaches and challenges in identifying clinically applicable cutoffs hamper their clinical utility but may allow identification of new biomarkers further improving risk prediction.

Despite these findings, the routine implementation of most inflammatory biomarkers remains limited, not only due to lack of large-scale studies investigating clinical usefulness, but also because of cost and logistical considerations.

### hsCRP and IL-6 - Key Inflammatory Biomarkers in HF

Among investigated inflammatory biomarkers, the majority of studies focused on hsCRP and IL-6 as clinically applicable inflammatory biomarkers for risk assessment showing strong and large-scale evidence of their predictive and prognostic utility in HF.

Studies analyzing hsCRP and IL-6 in individuals without HF have provided evidence on the predictive capacity for incident HF.

In the Multi-Ethnic Study of Atherosclerosis (MESA) study, elevated hsCRP levels in patients undergoing statin therapy were strongly associated with incident HF, all-cause mortality and provided incremental value for the prediction of HF events [86]. Burger et al. reported that in patients with established CVD, defined as coronary artery disease, cerebrovascular disease, peripheral artery disease and/or abdominal aortic aneurysm, but without preexisting HF, higher hsCRP levels were associated with an increased incidence of HFrEF and HFpEF [87]. These findings were confirmed by a systematic review and meta-analysis published in 2021, which demonstrated that higher levels of hsCRP were not only associated with higher risk of development of new onset HFpEF, but also with CV- and all-cause mortality in HFpEF patients, showing that hsCRP predicts incidence as well as adverse outcomes in established HFpEF [88].

To better understand the upstream role of IL-6 in the development of HF, a case-cohort study published in 2021 demonstrated a significant association between IL-6 and the development of HFpEF in the general population [89]. Furthermore, Khan et al. have performed further analyses in 6622 apparently healthy individuals included in the MESA study. The authors showed that elevated IL-6 levels were strongly predictive for mortality, adverse CV outcomes and incident HF [90]. Recent proteomic data published in 2024 highlight that IL-6 acts as a central inflammatory node in the pathogenesis of HHF, with sex-specific differences in inflammatory profiles and a stronger upregulation of immune-related pathways in women. This also highlights the potential value of sex-stratified biomarker approaches [91].

Beyond this, both biomarkers have been investigated in patient cohorts with established HF.

A systematic review from 2009 assessed the prognostic capacity of hsCRP in general and high-risk populations, showing that hsCRP is an independent predictor for incident HF but also adverse CV events in HF patients [92]. In 2020, a study in patients with chronic HF found that hsCRP is associated with all-cause mortality and CV mortality [93]. Furthermore, a recent subanalysis of the Treatment of Preserved Cardiac Function Heart Failure with an Aldosterone Antagonist (TOPCAT) trial subjects with HFpEF, those with hsCRP levels ≥ 2 mg/L had a higher rate of HHF before randomization and were linked to an increased risk of CV death and HF [94]. An analysis of the The BIOlogy Study to TAilored Treatment in Chronic Heart Failure (BIOSTAT-CHF) cohort in 2329 patients with an LVEF ≤ 40% demonstrated an independent relationship between elevated IL-6 levels and hard CV outcomes [95]. In HFpEF, similar associations between IL-6 and all-cause mortality, CV death and subsequent HHF have been observed in 286 individuals recently hospitalized with HFpEF [96]. These findings reinforce the role of inflammation as a critical residual risk factor in HF, emphasizing its importance in enhancing diagnostic accuracy and improving risk assessment.

### Head-to-head Comparison of hsCRP and IL-6

To better understand their clinical applicability, studies have directly compared IL-6 and hsCRP as central inflammatory biomarkers in HF. In 2003, a substudy of the Framingham Heart Study investigated subjects of older age without HF or prior MI. Here, elevated IL-6 levels were associated with a 68% increased risk of developing chronic HF and serum levels of CRP ≥ 5 mg/dL showed a 2.8-fold increased risk [97]. The Health-ABC study investigated the predictive capacity of TNF-⍺, IL-6 and CRP in 2610 elderly individuals without previous HF over a median follow-up of 9.4 years. IL-6 and CRP were associated with a 29% and 9% increased risk of development of HF. In a direct comparison to IL-6, CRP did not show multivariable stability in a model including all three biomarkers. Furthermore, the association of the biomarkers was more pronounced in individuals with an LVEF > 45% than in those with an LVEF ≤ 45%. Lastly, addition of IL-6 to the Health-ABC HF model demonstrated enhanced model performance, as evaluated with C-index and Bayes information criterion [98]. More recently, two analyses in apparently healthy individuals of the MESA study have shown that IL-6 and hsCRP were primarily associated with incidence of HFpEF but not HFrEF or HFmrEF [99]. Moreover, a head-to-head comparison of hsCRP and IL-6 revealed a higher rate of adverse events, including HF in those with elevated IL-6 levels irrespective of hsCRP levels [100].

However, in patients with established HF, studies directly comparing the prognostic capacity of IL-6 and hsCRP are scarce. A study in patients with stable chronic HF published in 2009 showed that IL-6 was an independent predictor for CV mortality and HHF, suggesting stronger predictive performance than hsCRP outperforming hsCRP (Adjusted hazard ratio (95% CI): IL-6: 2.74 (1.17, 6.43) vs. hsCRP: 1.07 (0.50, 2.32); Chi^2^: IL-6: 5.39 vs. hsCRP: 0.03) [101]. However, this small study only included 201 patients with heterogenous patient characteristics. Further evidence from 1086 HF patients in the Ludwigshafen Risk and Cardiovascular Health (LURIC) study shows that IL-6 independently predicts CV mortality in patients with HFpEF but not HFrEF whereas hsCRP was not significantly associated with the primary outcome after multivariable adjustment [102].

Taken together, available evidence shows that IL-6 and hsCRP both are associated with incident HF and adverse outcomes in established HF, highlighting their potential as biomarkers for risk stratification in HF. However, their potential clinical applicability differs strongly, and further comparative studies are needed to validate these findings and to clarify the clinical utility of each biomarker in routine care.

### Practical Aspects of Inflammatory Biomarkers Assessment in Clinical Routine

Despite strong evidence and various studies that have shown associations with relevant outcomes, neither hsCRP nor IL-6 has been established as a routine biomarker for risk stratification in HF.

**Fig. 3:**
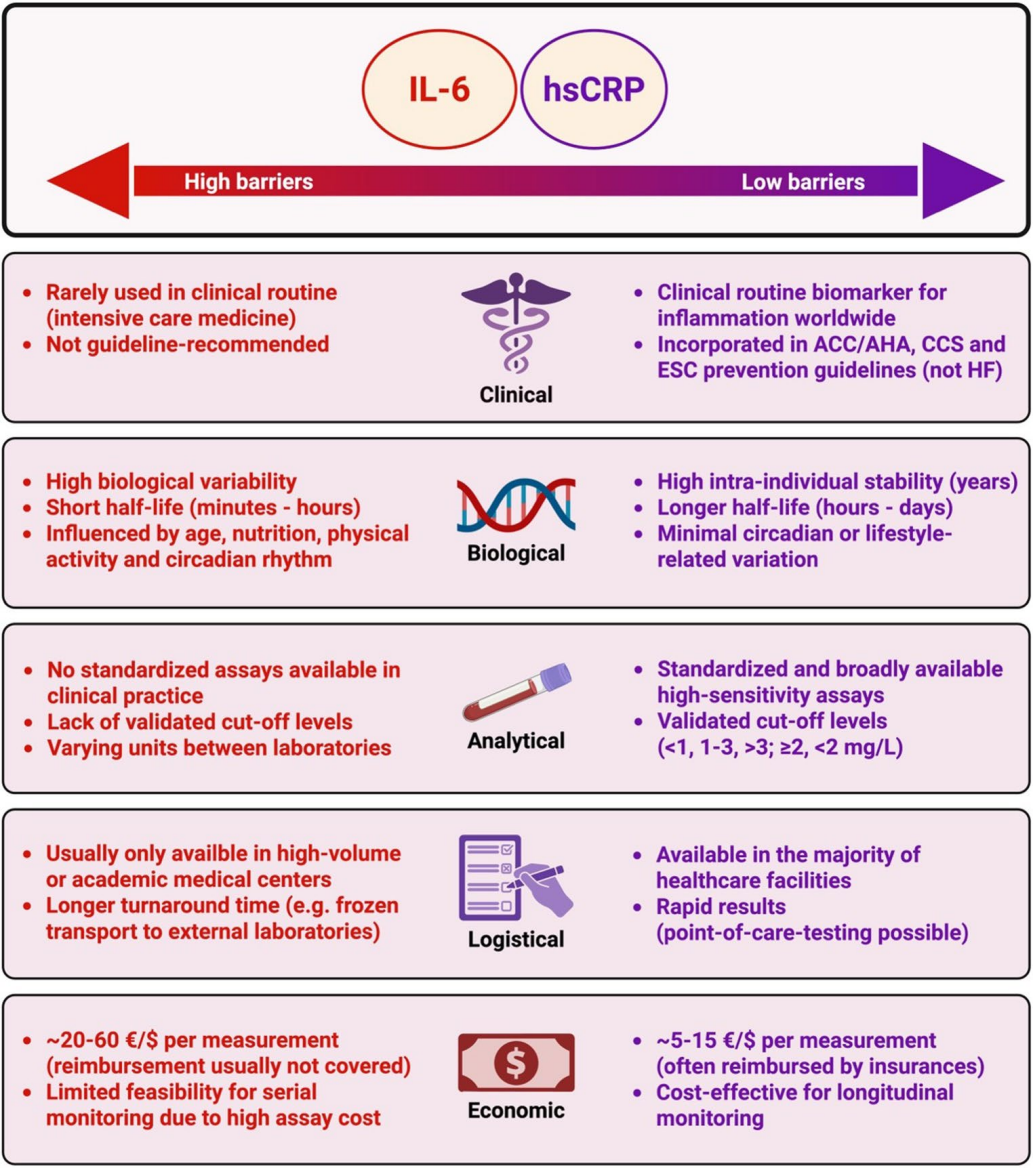
**Practical aspects of implementing inflammatory biomarkers in heart failure**. Comparison of IL-6 and hsCRP across five key domains relevant to clinical implementation: clinical utility, biological stability, assay availability, logistical feasibility, and economic aspects. While hsCRP is already guideline-endorsed in ASCVD-prevention, IL-6 remains limited by variability, lack of standardization, and higher costs. Both biomarkers have not been recommended in HF guidelines yet. Abbreviations: ACC = American College of Cardiology; AHA = American Heart Association; ASCVD = Atherosclerotic cardiovascular disease; CCS = Canadian Cardiovascular Society; ESC = European Society of Cardiology; hsCRP = High-sensitivity C-reactive protein; IL-6 = Interleukin-6 (Figure created with BioRender.com)

Barriers to the clinical implementation of IL-6 measurements in HF care can be linked to its biological variability, shorter half-life and influences of age, nutrition, physical activity and circadian rhythm as well as rare use in daily clinical routine [107–111]. In contrast, several aspects support the clinical use of hsCRP as a routinely applicable biomarker (Fig. 3).

First, hsCRP, broadly integrated in daily clinical practice globally, shows strong intra-individual stability, longer half-life as well as notably lower measuring costs (typically 5–15 € or USD), which is approximately three to four times less than IL-6 testing in Germany and the US [112, 113]. Second, concentrations of hsCRP are not influenced by time-of-day variation compared to IL-6 [114]. Importantly, measurement of inflammatory biomarkers for CV risk assessment, particularly hsCRP and IL-6, should only be performed in a clinically stable state, as acute inflammation or other secondary causes may significantly alter their levels [115–117]. However, hsCRP levels remain stable in serial measurements if testing is performed outside of acute illness [113, 118]. Third, assessment of hsCRP levels for risk stratification is already partially included in global guidelines for primary and secondary prevention of ASCVD, whereas IL-6 measurements have not been guideline-recommended yet [55, 103–105]. Fourth, standardization of IL-6 measurement remains a major limitation, whereas in current clinical standards, high-sensitivity assays are widely available for CRP assessment, which are necessary to assess hsCRP levels attributed to low-grade systemic inflammation [118]. Fifth, current IL-6 assays lack validated cut-off levels limiting clinical use. For hsCRP, cut-off points have been established in observational studies and clinical trials. In the JUPITER and CANTOS trials, systemic low-grade inflammation has been defined by hsCRP levels ≥ 2 mg/L [12, 13]. A more granular stratification into hsCRP levels of < 1 mg/L, 1–3 mg/L and > 3 mg/L provides further clinically useful cutpoints indicating low, average, or high residual inflammatory risk [112, 119, 120]. Lastly, IL-6 levels may increase with certain anti-inflammatory therapies, potentially causing confusion in clinical routine. In summary, direct comparisons indicate that hsCRP is a practical biomarker for routine use, whereas IL-6 screening is unlikely to be adopted in standard practice. Nevertheless, given its likely causal role, targeting inflammation through the IL-6 pathway in HF remains a promising strategy, which will be discussed in the following paragraph.

### Anti-inflammatory Therapy in Heart Failure

Previous and ongoing clinical trials highlight the potential of anti-inflammatory therapies in CVD, specifically ASCVD and HF (Table 2). However, further research is required to integrate these therapeutic approaches into clinical practice. Nevertheless, inflammatory biomarkers have the potential to guide therapeutic allocation and monitoring. This is especially relevant in the context of anti-inflammatory therapies targeting upstream inflammatory pathway activation and aiming to improve clinical outcomes. Prior large-scale evidence has highlighted the role of inflammation and corresponding biomarkers in primary and secondary prevention of ASCVD. A substudy of CANTOS demonstrated that a reduction of hsCRP levels < 2 mg/L achieved by canakinumab treatment showed a higher prognostic benefit for HHF and HF-related CV mortality than residual hsCRP levels of ≥ 2 mg/L after treatment [121].

**Table 2:** Clinical trials with anti-inflammatory therapeutics in ASCVD and HF

Trial	Target/therapeutic	Population	n*	Primary endpoint	Result
**Patients with ASCVD**					
Canakinumab Anti-inflammatory Thrombosis Outcome Study (**CANTOS**) [13]	IL-1β/canakinumab	Individuals with prior MI and hsCRP ≥ 2 mg/dL	10,061	3PMACE	Reduction
Anti-Inflammatory Therapy with Canakinumab for the Prevention of Hospitalization for Heart Failure [121]	IL-1β/canakinumab	Post-MI patients with hsCRP ≥ 2 mg/L	10,061	HHF and the composite of HHF or HF–related mortality	Reduction
Colchicine in Patients with Chronic Coronary Disease (**LoDoCo2**) [14]	Inflammasome/colchicine	Clinically stable (> 6 months) individuals with angiographically confirmed CAD	5522	Composite of CV death, spontaneous (nonprocedural) MI, ischemic stroke, or ischemia-driven coronary revascularization	Reduction
Efficacy and Safety of Low-Dose Colchicine after Myocardial Infarction (**COLCOT**) [132]	Inflammasome/colchicine	MI within the last 30 days, treated according to national guidelines with completed PCI	4745	Composite of CV death, resuscitated cardiac arrest, MI, stroke, or urgent hospitalization for angina leading to coronary revascularization.	Reduction
CoLchicine and spironolactonE in patients with ST elevation myocARdial infarction/SYNERGY Stent Registry – Organization to Assess Strategies for Ischemic Syndromes 9 (**CLEAR-SYNERGY (OASIS 9)**) [15]	Inflammasome/colchicine	MI within the last 72 h	7062	Composite of CV death, recurrent MI, stroke, or unplanned ischemia-driven coronary revascularization	No reduction
A Research Study to Look at How Ziltivekimab Works Compared to Placebo in People With Cardiovascular Disease, Chronic Kidney Disease and Inflammation (**ZEUS**) (NCT05021835)	IL-6/ziltivekimab	Individuals with evidence of ASCVD (CAD, CVD or PAD), hsCRP ≥ 2 mg/L and CKD (eGFR: 15–59 mL/min or UACR ≥ 200 mg/g with eGFR ≥ 60 ml/min)	(6200)	3PMACE	Ongoing
A Research Study to Look at How Ziltivekimab Works Compared to Placebo in People With a Heart Attack (**ARTEMIS**) (NCT06118281)	IL-6/ziltivekimab	Type 1 MI (angiographically confirmed), STEMI or NSTEMI, plus ≥ 1 high-risk feature: prior MI/revascularization, diabetes, CKD, ischemic stroke, carotid/PAD, or multivessel CAD.	(10,000)	3PMACE	Recruiting
A Phase 2b/3, Multicenter, Randomized, Double-Blind, Placebo- Controlled, Combined Dose-Finding and Cardiovascular Outcome Study to Investigate the Efficacy and Safety of CSL300 (Clazakizumab) in Subjects with End Stage Kidney Disease Undergoing Dialysis (CSL300_2301) (**POSSIBLE6ESKD**) (NCT05485961) [133]	IL-6/clazakizumab	Individuals with evidence of ASCVD or diabetes mellitus, hsCRP ≥ 2 mg/L and a diagnosis of end stage kidney disease undergoing maintenance dialysis for at least 12 weeks	(2310)	Change from Baseline hs-CRP (Phase 2b) Composite of CV death or MI (Phase 3)	Phase 2b (completed): Up to 92% reduction of hsCRP after 12 weeks Phase 3: recruiting
**Patients with chronic HF**					
Targeted anticytokine therapy in patients with chronic heart failure: results of the Randomized Etanercept Worldwide Evaluation (**RENEWAL**) [122]	TNF-α/etanercept	HF NYHA II-IV, LVEF ≤ 30%	1673	Composite of all-cause mortality or HHF	No reduction
Randomized, double-blind, placebo-controlled, pilot trial of infliximab, a chimeric monoclonal antibody to tumor necrosis factor-alpha, in patients with moderate-to-sever heart failure: results oft the anti-TNF Therapy Against Congestive Heart Failure (**ATTACH**) Trial [123]	TNF-α/infliximab	HF NYHA III-IV, LVEF ≤ 35%	150	All-cause mortality, HHF, change in NYHA class	No reduction
Effects of interleukin-1 blockade with anakinra on aerobic exercise capacity in patients with heart failure and preserved ejection fraction (from the **D-HART** pilot study) [124]	IL-1/anakinra	HFpEF	12	Aerobic exercise capacity	Improvement
IL-1 Blockade in Patients with Heart Failure With Preserved Ejection Fraction (**D-HART 2**) [125]	IL-1/anakinra	HFpEF	31	Peak oxygen consumption (VO_2_ max)	No reduction
Safety and Tolerability Study of AZD4831 in Patients With Heart Failure (**SATELLITE)** [134]	Myeloperoxidase/mitiperstat	HFmrEF and HFpEF	41	MPO specific activity and safety (Phase 2a)	75% decrease in placebo-adjusted MPO activity
Study to Evaluate the Efficacy and Safety of AZD4831 in Participants With Heart Failure With Left Ventricular Ejection Fraction > 40% (**ENDEAVOR**) (NCT04986202) [135, 136]	Myeloperoxidase/mitiperstat	HFmrEF and HFpEF	711	KCCQ and 6MWD	No improvement
Effects of Ziltivekimab Versus Placebo On Morbidity And Mortality in Patients With Heart Failure With Mildly Reduced Or Preserved Ejection Fraction And Systemic Inflammation (**HERMES**) (NCT05636176) [130]	IL-6/ziltivekimab	HFmrEF and HFpEF, hsCRP > 2 mg/L	(5600)	Composite of CV-death, HHF, or urgent HF visit	Ongoing
A Research Study Looking at How Ziltivekimab Works Compared to Placebo in Participants With Heart Failure and Inflammation (**ATHENA**) (NCT06200207)	IL-6/ziltivekimab	LVEF > 40% within last 12 months, with no interim cardiac event and hsCRP ≥ 2 mg/L	(680)	Change in KCCQ-CSS	Recruiting

Selected clinical trials evaluating anti-inflammatory therapies in patients with ASCVD and HF. Trials are grouped by target population, therapeutic agent, and inflammatory pathway, with summary primary outcomes reported

* = estimated study sample after completion of enrollment.

Abbreviations: *3PMACE *3-point major adverse cardiovascular events (non-fatal myocardial infarction, non-fatal stroke, and cardiovascular death), *6MWD* 6-minute walking distance, *ASCVD* Atherosclerotic cardiovascular disease, *CAD* Coronary artery disease, *CKD* Chronic kidney disease, *CVD* Cerebrovascular disease, *CV* Cardiovascular, *eGFR* Estimated glomerular filtration rate, *HF* Heart Failure, *HHF* Hospitalization for heart failure, *HFmrEF* Heart failure with mildly reduced ejection fraction, *HFpEF* Heart failure with preserved ejection fraction, *hsCRP* High-sensitivity C-reactive protein, *IL-1* Interleukin-1, *IL-1β* Interleukin-1 beta, *IL-6* Interleukin-6, *KCCQ-CSS* Kansas City Cardiomyopathy, *KCCQ-CSS* Kansas City Cardiomyopathy Questionnaire clinical summary score, *LVEF* Left ventricular ejection fraction, *MI* Myocardial infarction, MPO Myeloperoxidase, *NSTEMI* Non-ST-elevation myocardial infarction, *NT-proBNP* N-terminal pro–B-type natriuretic peptide, *NYHA* New York Heart Association, *PAD* Peripheral artery disease, *PCI* Percutaneous coronary intervention, *STEMI* ST-elevation myocardial infarction, *TNF-α* Tumor necrosis factor-alpha, *UACR* Urinary albumin-to-creatinine ratio, *VO₂* max Peak oxygen consumption

The potential effect of anti-inflammatory therapies on symptoms and outcomes in patients with chronic HF has been examined in several clinical trials. The Randomized EtaNErcept Worldwide evALuation (RENEWAL) trial evaluated the TNF-α antagonist etanercept in patients with HF and New York Heart Association (NYHA) class II-IV with a LVEF ≤ 30%. After 96 weeks, no significant differences in event-free survival were observed between the intervention and placebo groups [122]. In the ATTACH (Anti-TNF Therapy Against Congestive Heart failure) trial, investigating infliximab, another TNF-α inhibitor, in patients with stable NYHA class III-IV HF and an LVEF ≤ 35%, subjects receiving infliximab had a higher rate of death from any cause or HHF, raising concerns about the use of TNF-α inhibitors in HF [123].

The central involvement of IL-6 in inflammatory pathways has prompted growing interest in its therapeutic modulation in HF. Inhibition of IL-6 signaling with the receptor antagonist anakinra has been investigated in the DHART and DHART-2 trials [124, 125]. The DHART trial, published in 2014, evaluated its effects in patients with HFpEF, reporting improvements in aerobic exercise capacity [124]. The follow-up DHART-2 trial demonstrated that anakinra significantly reduced levels of hsCRP and NT-proBNP in obese HFpEF patients. However, it neither improved peak oxygen consumption (VO_2_ max) nor enhanced the oxygen uptake efficiency slope [125]. Rheumatoid arthritis (RA), as a systemic (auto-)inflammatory disease, is associated with subclinical cardiac involvement and is a model disease for studying inflammation-mediated cardiac effects [126, 127]. Studies have shown that in women with RA and no prior clinically apparent cardiac disease, IL-6 inhibition with tocilizumab improves LVEF and reduces left ventricular mass index, with cardiac magnetic resonance imaging confirming normalization of left ventricular morphology [128]. Additionally, in patients with RA and no evidence of clinical HF, tocilizumab treatment has been associated with reductions in NT-proBNP levels (median 42.5 pg/mL vs. 109.0 pg/mL, *p* < 0.001), suggesting a potential early benefit in alleviating inflammation-related cardiac stress prior clinical manifestation of HF [129].

The cumulative evidence provided the rationale for IL-6 to be continuously considered as a key drug target and as a tool for guiding treatment success of targeted anti-inflammatory therapies, specifically in HF. Clinical trials are currently investigating targeted approaches in HF subtypes, that are primarily driven by systemic low-grade inflammation. Among the ongoing trials, the HERMES trial (NCT05636176) investigates the use of a human monoclonal antibody targeting the IL-6 ligand in patients with HFmrEF and HFpEF who present with elevated residual inflammation, defined by hsCRP levels ≥ 2 mg/L at enrollment [130, 131].

### Future Perspectives

**Fig. 4:**
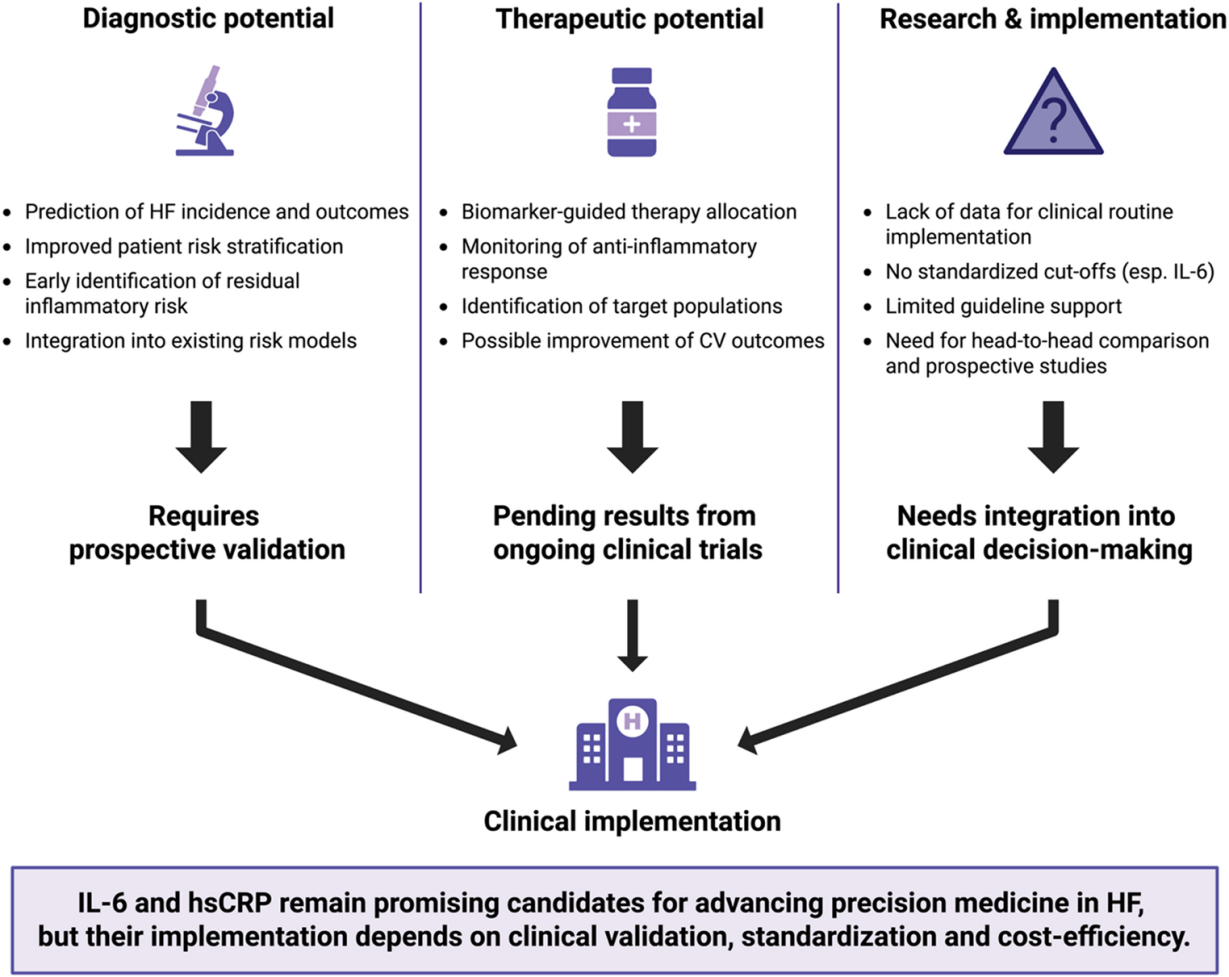
**The future of inflammatory biomarkers in heart failure care: From potential to clinical implementation**. Summary of the diagnostic, therapeutic, and implementation-related potentials of inflammatory biomarkers in HF. While IL-6 and hsCRP hold promise for improving risk prediction and guiding anti-inflammatory strategies, clinical implementation requires prospective validation, standardization of cut-offs, and integration into routine care pathways. Abbreviations: CV = Cardiovascular; HF = Heart failure; hsCRP = High-sensitivity C-reactive protein; IL-6 = Interleukin-6 (Figure created with BioRender.com)

Further research is required to clarify the practical benefits of inflammatory biomarkers for HF management (Fig. 4). From a diagnostic perspective, inflammatory biomarkers improve identification and stratification of patients with- or at-risk of HF. Therapeutically, they potentially assist in guiding treatment decisions and monitoring the response of novel interventions, such as anti-inflammatory therapies. Anti-inflammatory therapies may potentially contribute to lowering symptom burden, improving CV outcomes and reducing healthcare costs in selected HF populations if ongoing trials provide sufficient evidence. IL-6 and hsCRP remain among the leading candidates for advancing precision medicine in HF. While hsCRP offers strong prognostic performance, established cut-offs, and broad clinical applicability, IL-6 provides a closer link to the underlying biology and may represent a modifiable therapeutic target. Large-scale, head-to-head comparisons are needed to assess how hsCRP and IL-6 could be combined to improve patient care and prospective studies should further clarify their impact on clinical decision-making and outcomes.

## Conclusion

Inflammation plays a central role in the development and progression of HF. Inflammatory biomarkers, particularly hsCRP and IL-6, are consistently associated with incident HF and adverse outcomes in established disease across diverse populations. They hold promise for improving HF care through refined risk stratification and therapeutic guidance. While their mechanistic and prognostic relevance are increasingly supported by clinical and genetic evidence, their routine integration into HF management and guidelines remains limited. While hsCRP is a strong biomarker candidate for clinical implementation, IL-6 is strongly linked to HF biology and represents a promising therapeutic target. Demonstrating the incremental value of inflammatory biomarkers within clinical algorithms and treatment monitoring will be essential for enabling more precise and individualized HF care.

## Key References

- Ridker PM, Everett BM, Thuren T, MacFadyen JG, Chang WH, Ballantyne C, et al. Anti-inflammatory Therapy with Canakinumab for Atherosclerotic Disease. N Engl J Med. 2017;377(12):1119-31.
  - A randomized clinical trial demonstrating that anti-inflammatory therapy reduced CV events in patients with previous myocardial infarction.
- Murphy SP, Kakkar R, McCarthy CP, Januzzi JL, Jr. Inflammation in Heart Failure: JACC State-of-the-Art Review. J Am Coll Cardiol. 2020;75(11):1324-40. 
  - This manuscript outlines the contribution of systemic inflammation to the development and progression of heart failure.
- Libby P. Targeting Inflammatory Pathways in Cardiovascular Disease: The Inflammasome, Interleukin-1, Interleukin-6 and Beyond. Cells. 2021;10(4). 
-
  - This paper reviews key inflammatory pathways in cardiovascular disease and the clinical potential of targeting these pathways for treatment.
- Libby P, Nahrendorf M, Swirski FK. Leukocytes Link Local and Systemic Inflammation in Ischemic Cardiovascular Disease: An Expanded “Cardiovascular Continuum”. J Am Coll Cardiol. 2016;67(9):1091 − 103. 
  - This paper shows how leukocytes connect systemic inflammation to ischemic heart disease, through an multi-organ network.
- Chia YC, Kieneker LM, van Hassel G, Binnenmars SH, Nolte IM, van Zanden JJ, et al. Interleukin 6 and Development of Heart Failure With Preserved Ejection Fraction in the General Population. J Am Heart Assoc. 2021;10(11):e018549.
  - This study describes the role of IL-6 in the development of heart failure.
- Mooney L, Jackson CE, Adamson C, McConnachie A, Welsh P, Myles RC, et al. Adverse Outcomes Associated With Interleukin-6 in Patients Recently Hospitalized for Heart Failure With Preserved Ejection Fraction. Circ Heart Fail. 2023;16 [4]:e010051. 
  - The paper showed that elevated IL-6 levels are predictive of adverse cardiovascular events.
- Cainzos-Achirica M, Miedema MD, McEvoy JW, Cushman M, Dardari Z, Greenland P, et al. The prognostic value of high sensitivity C-reactive protein in a multi-ethnic population after > 10 years of follow-up: The Multi-Ethnic Study of Atherosclerosis (MESA). Int J Cardiol. 2018;264:158 − 64. 
  - This manuscript demonstrated the prognostic role of hsCRP.
- Ridker PM, Rane M. Interleukin-6 Signaling and Anti-Interleukin-6 Therapeutics in Cardiovascular Disease. Circ Res. 2021;128(11):1728-46. 
  - This paper reviews the role of IL-6 in cardiovascular disease and highlights IL-6 inhibition as a promising therapeutic approach.

## Data Availability

No datasets were generated or analysed during the current study.
